# Stable Levels of Antibodies Against Unrelated Toxoid Vaccines After COVID-19: COVID-19 Infection Does Not Affect Toxoid Vaccine Antibody Levels

**DOI:** 10.20411/pai.v8i2.627

**Published:** 2024-02-07

**Authors:** Suvi T. Jokiranta, Simo Miettinen, Sami Salonen, Lauri Kareinen, Ruut Uusitalo, Essi M. Korhonen, Jenni Virtanen, Ilkka Kivistö, Kirsi Aaltonen, Dina A. Mosselhy, Tinja Lääveri, Anu Kantele, T. Petteri Arstila, Hanna Jarva, Olli Vapalahti, Santtu Heinonen, Eliisa Kekäläinen

**Affiliations:** 1 Department of Bacteriology and Immunology, University of Helsinki, Helsinki, Finland; 2 Translational Immunology Research Program, Faculty of Medicine, University of Helsinki, Helsinki, Finland; 3 Viral Zoonosis Research Unit, Medicum, Department of Virology, University of Helsinki, Helsinki, Finland; 4 Department of Veterinary Biosciences, University of Helsinki, Helsinki, Finland; 5 HUS Diagnostic Center, Clinical Microbiology, Helsinki University Hospital, Helsinki, Finland; 6 Department of Virology, Medicum, Faculty of Medicine, University of Helsinki, Helsinki, Finland; 7 Finnish Food Authority, Helsinki, Finland; 8 Microbiological Unit, Fish Diseases Department, Animal Health Research Institute, ARC, Dokki, Giza 12618, Egypt; 9 Infectious Diseases, Inflammation Center, University of Helsinki and Helsinki University Hospital, Helsinki, Finland; 10 Aalto University, Department of Computer Science, School of Science, Espoo, Finland; 11 Human Microbiome Research Program, Faculty of Medicine, University of Helsinki, Helsinki, Finland; 12 Meilahti Infectious Diseases and Vaccine Research Center, MeiVac, Department of Infectious Diseases, University of Helsinki, Helsinki, Finland; 13 New Children's Hospital, Pediatric Research Center, University of Helsinki and Helsinki University Hospital, Helsinki, Finland

**Keywords:** COVID-19, Vaccines, Antibodies, Post-acute COVID-19 syndrome, Immunity, Humoral

## Abstract

**Background:**

Lymphopenia is common in COVID-19. This has raised concerns that COVID-19 could affect the immune system akin to measles infection, which causes immune amnesia and a reduction in protective antibodies.

**Methods:**

We recruited COVID-19 patients (n = 59) in Helsinki, Finland, and collected plasma samples on 2 to 3 occasions during and after infection. We measured IgG antibodies to diphtheria toxin, tetanus toxoid, and pertussis toxin, along with total IgG, SARS-CoV-2 spike protein IgG, and neutralizing antibodies. We also surveyed the participants for up to 17 months for long-term impaired olfaction as a proxy for prolonged post-acute COVID-19 symptoms.

**Results:**

No significant differences were found in the unrelated vaccine responses while the serological response against COVID-19 was appropriate. During the acute phase of the disease, the SARSCoV-2 IgG levels were lower in outpatients when compared to inpatients. SARS-CoV-2 serology kinetics matched expectations. In the acute phase, anti-tetanus and anti-diphtheria IgG levels were lower in patients with prolonged impaired olfaction during follow up than in those without.

**Conclusions:**

We could not detect significant decline in overall humoral immunity during or after COVID-19 infection. In severe COVID-19, there appears to be a temporary decline in total IgG levels.

## INTRODUCTION

The pandemic SARS-CoV-2 virus has caused more than 6.9 million deaths worldwide since 2019 [1]. In moderate-to-severe COVID-19, lymphopenia is a cardinal finding with lower lymphocyte counts associated with more severe disease [2]. This lymphopenia is evident in both T-cell and B-cell populations [3], raising concerns about the possible long-term effects of COVID-19 infection on the immune system. Immune amnesia, where an infection can erase acquired immunity to other pathogens/antigens, has been previously shown after measles [4, 5]. Measles infection depletes memory and naive B cells [5], which causes a drop in circulating antibody levels and reversal to immunological immaturity [4, 5]. Previous research has shown decreases in antibodies against routine vaccines during and/or after COVID-19 infection [6–8]. However, the results have been inconclusive, and no follow up has been conducted. To date, no studies have been conducted on routine vaccine antibodies in patients exhibiting post-acute sequelae of COVID-19 (PASC; colloquially known as long COVID).

Immunological amnesia may remain of no clinical significance, but it has the potential to lead to unexpected consequences. Immune amnesia may render the individual again susceptible to some vaccine-preventable diseases and, theoretically at the population level, even lead to epidemics [9]. There is also evidence that post-acute sequelae of COVID-19 are associated with persisting inflammation and immune dysregulation [10]. Based on our previous work concerning heterologous boosting of unrelated immunity in Puumala hantavirus infection [11], we studied the effect of SARS-CoV-2 infection on pre-existing vaccine responses. Additionally, we examined the relationship of PASC (as defined by impaired olfaction) to the vaccine responses.

We chose to study antibodies against toxoid vaccines: in Finland, diphtheria, tetanus, and pertussis vaccination primary series coverage is >95%. In the Finnish national vaccination program, booster doses of toxoid vaccines are currently given at 20-year intervals (for those aged ≥ 65 at 10-year intervals) or not at all (pertussis) after the age of 25 years [12]. Thus, there is a low chance of patients receiving a booster in the follow-up period. We, therefore, reasoned that studying the antibody levels against toxoid vaccines during and after COVID-19 can be used to exclude significant immunological amnesia caused by the SARS-CoV-2 virus.

## METHODS

### Patient Cohort

Patients positive for SARS-CoV-2 reverse transcription polymerase chain reaction (RT-PCR) aged 24 to 81 years (n = 59), either admitted to Helsinki University Hospital in Helsinki, Finland, (n = 22) or treated as outpatients (n = 37), were recruited to this study. The patients were recruited between March 2020 and February 2021. During this time, there were 2 major waves of infections caused by the original Wuhan strain. The Alpha variant was first detected in December 2020 in Finland, and its prevalence rapidly increased in early 2021 [13]. Therefore, we conclude that the majority of cases studied represent infections of the Wuhan strain with a minor fraction of Alpha variant infections. None of the patients studied had received COVID-19 vaccines prior to infection. None of the patients tested positive for COVID-19 reinfection in the study period.

Peripheral blood samples were collected from each individual at 2 or 3 different times during and after infection. The sampling times differed between the patients. Because of this, the samples were divided between 3 disease phases for the sake of data analysis: acute (0 to 35 days after symptom onset), convalescent (36 to 120 days), and recovered (over 120 days after symptom onset). Later, these will be referred to as A, B, and C phases.

The study was approved by the Ethics Committee of the Hospital District of Helsinki and Uusimaa (HUS/853/2020, HUS/1238/2020). All participants gave written informed consent in accordance with the Declaration of Helsinki.

### Plasma and Serum Samples

Plasma and serum were separated from the blood samples by centrifugation, and the samples were stored at −80°C for later use. Upon use, the frozen samples were thawed at room temperature.

### Vaccine Serology

IgG antibodies against Diphtheria Toxin were measured using Novagnost® Diphtheria Toxin 5S IgG (NovaTec Immunodiagnostica GmbH, Dietzenbach, Germany). IgG antibodies against Tetanus Toxoid were measured using Anti-Tetanus Toxoid ELISA (IgG) (EUROIMMUN, Lübeck, Germany), and IgG antibodies against Bordetella pertussis toxin were measured using Anti-Bordetella pertussis toxin ELISA (IgG) (EUROIMMUN). The samples were analyzed in the HUS Diagnostic Center Clinical Microbiology, Helsinki, Finland, with a fully automatic EuroLabWorkstation ELISA (EUROIMMUN), and the analysis was carried out according to the manufacturer's instructions.

### Total IgG

Total IgG was determined at the clinical diagnostic laboratory HUS Diagnostic Center Clinical Chemistry Facility in Helsinki, Finland, using the clinically accredited Siemens Atellica CH IgG_2 assay.

### SARS-CoV-2 Serology

SARS-CoV-2 spike (S) protein ELISA was performed as previously described by Rusanen et al [14]. SARS-CoV-2 neutralizing antibody assay was performed as previously described by Haveri et al [15].

### Persisting Symptoms

We chose to use impaired olfaction as a proxy of persisting symptoms after acute COVID-19, as it has been most commonly associated with PASC. Moreover, unlike many other symptoms, such as fatigue, it is uncommon among SARS-CoV-2-negative individuals [16–18]. The data on persisting symptoms used here were obtained from the Long Covid Master study [16] with 1326 participants whose symptoms were evaluated with an online questionnaire.

### Statistical Analysis

All analyses were performed using R Statistical Software (v4.2.0; R Core Team 2022). Friedman, Wilcoxon, and Mann-Whitney U tests as well as *t* tests via the rstatix R package (v0.7.0; Kassambara A 2021) were employed to compare differences between groups. Figures were created via the gg-plot2 R package (v3.3.6; Wickham H 2016). The limit for statistical significance was set to *P* < 0.05.

## RESULTS

The patient demographic data are summarized in Table 1. Most patients were sampled at all 3 disease phases (acute, convalescent, and recovered), but we had to exclude 4 patients because all their samples fell within the same phase, which prevented meaningful comparisons. There were 12 patients who had samples within 2 different phases, and these had to be excluded from the non-parametric paired multiple comparisons tests (Friedman test), requiring a complete block design. They are, however, included in the figures and the nonparametric pairwise comparisons (Wilcox-on tests). The median time from symptom onset to sampling is presented in Table 1 where it can be seen that, on average, in the acute phase inpatients were sampled earlier than outpatients.

**Table 1.** Demographics

	**Outpatients**		**Inpatients**		**All**	
	***n***	***%***	***n***	***%***	***n***	***%***
**N**	37	62.7	22	37.3	59	100.0
**Sex**						
Male	15	40.5	10	38.5	25	39.7
Female	22	59.5	16	61.5	38	60.3
**Age**						
18-29	4	10.8	0	0	4	6.8
30-39	10	27.0	1	4.5	11	18.6
40-49	6	16.2	3	13.6	9	15.3
50-59	6	16.2	9	40.9	15	25.4
60-69	7	18.9	7	31.8	14	23.7
70-79	3	8.1	2	9.1	5	8.5
80-	1	2.7	0	0	1	1.7
*Median*	*45*		*58*		*53*	
**Days in hospital**						
0	37	100.0			37	62.7
1-7			7	31.8	7	11.9
8-14			7	31.8	7	11.9
15-21			5	22.7	5	8.5
22 -			3	13.7	3	5.0
*Median*	*0*		*10.5*		*0*	
**Number of patients with 3 samples in different phases**						
Vaccine IgG	33	89.2	10	45.5	43	72.9
Total IgG	32	86.5	10	45.5	42	71.2
Neutr. titer	30	81.1	1	4.5	31	52.5
S protein IgG	32	86.5	10	45.5	42	71.2
**PASC**						
Yes	11	29.7	5	22.7	16	27.1
No	22	59.5	11	50.0	33	55.9
No data	4	10.8	6	27.3	10	16.9
	**Outpatients**		**Inpatients**		**All**	
	***med.***	***min/max***	***med.***	***min/max***	***med.***	***min/max***
**Sample times (days from symptom outset)**						
Acute phase (A)	25.5	9/34	11	7/24	21	7/34
Convalescent p. (B)	91.5	61/108	98.5	57/112	95	57/112
Recovered p. (C)	156	123/239	375	206/417	166	123/417

**Figure 1.**
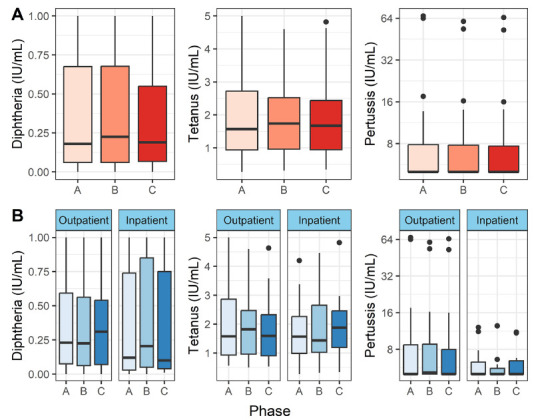
**Diphtheria, tetanus, and pertussis toxoid antibodies in peripheral blood.** Vertical lines denote the range of minimum and maximum values, the box denotes 1st and 3rd quartile values, and the middle horizontal line denotes the median value. Black dots denote outlier values. A = acute (n = 55), B = convalescent (n = 50), C = recovered (n = 48). Figure 1A shows the distributions in all patients, and 1B shows outpatients and hospitalized patients separately.

To test our main hypothesis of COVID-19-induced immunological amnesia, we analyzed antibodies against diphtheria, tetanus, and pertussis toxoid/toxin. Figure 1 shows an overview of the results. We found no statistically significant differences between the disease phases in either the entire patient population (*P* > 0.05, Friedman test) or only in the inpatients (*P* > 0.05, paired Wilcoxon test).

**Figure 2.**
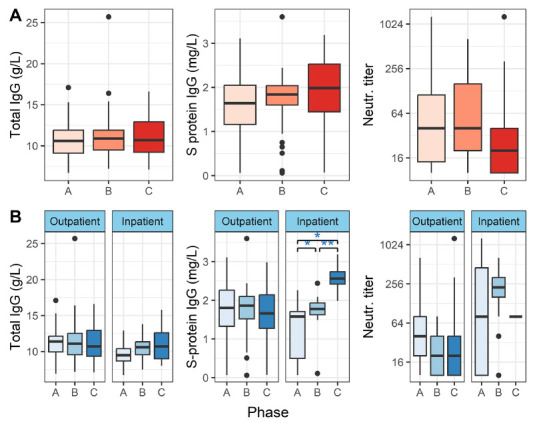
**Total IgG, SARS-CoV-2 Spike protein IgG, and SARS-CoV-2 neutralizing antibody titers in peripheral blood.** Vertical lines denote the range of minimum and maximum values, the box denotes 1st and 3rd quartile values, and the middle horizontal line denotes the median value. Black dots denote outlier values. A = acute, B = convalescent, C = recovered. Significance: * *P*< 0.05, ** *P* < 0.01 (paired Wilcoxon test). Figure 1A shows the distributions in all patients, and 1B shows outpatients and inpatients separately.

It is known that total IgG may wane in severe infection, and this could affect the specific IgG levels against toxoid vaccines shown in Figure 1. To account for this effect, we analyzed total IgG levels (Figure 2). We found no significant changes in total IgG levels in either outpatients or inpatients (*P* > 0.05, Friedman and Wilcoxon paired tests).

Additionally, we determined SARS-CoV-2 S-protein IgG and neutralizing antibody titers (Figure 2). We could only determine neutralizing antibody titer at the C phase from 1 inpatient, therefore we excluded the C phase from further analysis in the subgroup of inpatients.

When all patients were included in the analysis, we could not show differences between the disease phases (*P* > 0.05, Friedman test). However, for inpatients, the S protein IgG levels increased with time (Figure 2B), and the levels differed significantly between all phases: A/B (*P* adj = 0.049), A/C (*P* adj = 0.008) and B/C (*P* adj = 0.006), as measured by the paired Wilcoxon test. In the inpatient group, neutralizing antibody titers did not differ significantly between the phases (*P* > 0.05, paired Wilcoxon test).

**Figure 3.**
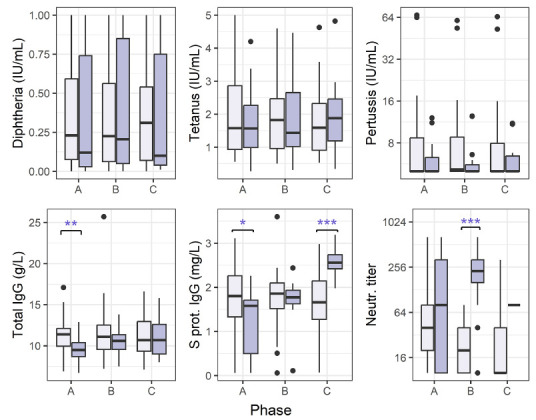
**Antibody levels for COVID-19 outpatients and hospitalized patients.** Vertical lines denote the range of minimum and maximum values, the box denotes 1st and 3rd quartile values, and the middle horizontal line denotes the median value. Black dots denote outlier values. Outpatients (light purple bars) and hospitalized (dark purple), phases A = acute, B = convalescent, C = recovered. Significance: * *P*< 0.05, ** *P* < 0.01, *** *P* < 0.001 (Mann-Whitney U test).

We were interested in the differences between the 2 groups of patients (outpatients and inpatients) (Figure 3). There were several statistically significant differences as measured by the non-parametric unpaired comparisons test (Mann-Whitney U test): for inpatients, total IgG was lower in the acute phase (*P* = 0.0032), S protein IgG lower in the acute (*P* = 0.0214) and higher in the recovered (*P* < 0.0001) phases, and neutralizing antibody titers were higher in the convalescent (*P* < 0.0001) phase.

Finally, we performed a subanalysis on those patients (n = 49) who had self-reported survey data on PASC symptoms. We compared the group that had reported impaired olfaction at 3 to 17 months after the initial positive SARS-CoV-2-PCR test (PASC+, n = 16) with the group that had reported no prolonged change in sense of smell (PASC-, n = 33) separately in all 3 phases. In the acute phase, there was a significant difference between PASC+ and PASC− patients in anti-tetanus IgG (*P* = 0.0067, unpaired Wilcoxon test) and anti-diphtheria IgG (*P* = 0.0155). Additionally, anti-tetanus IgG showed a significant difference also in the convalescent phase (*P* = 0.0250). In all cases, the anti-toxoid vaccine IgG levels were lower in the PASC+ group. Anti-pertussis IgG did not differ significantly between the groups, and neither did total IgG levels or SARS-CoV-2 serology.

To assess for confounding factors, we examined the PASC+ and PASC− groups more closely. The PASC+ group was, on average, younger (mean 46.6 years) than the PASC− group (mean 56.2 years), with a significant difference between the groups (*P* = 0.0369, 2-sample *t* test). However, there was no significant correlation between patient age and vaccine responses (*P* > 0.05, Spear-man correlation, data not shown). The PASC+ and PASC− groups had no significant differences in any other tested variable: sex, hospitalization status, length of hospitalization, and time from symptom onset to sample collection.

**Figure 4.**
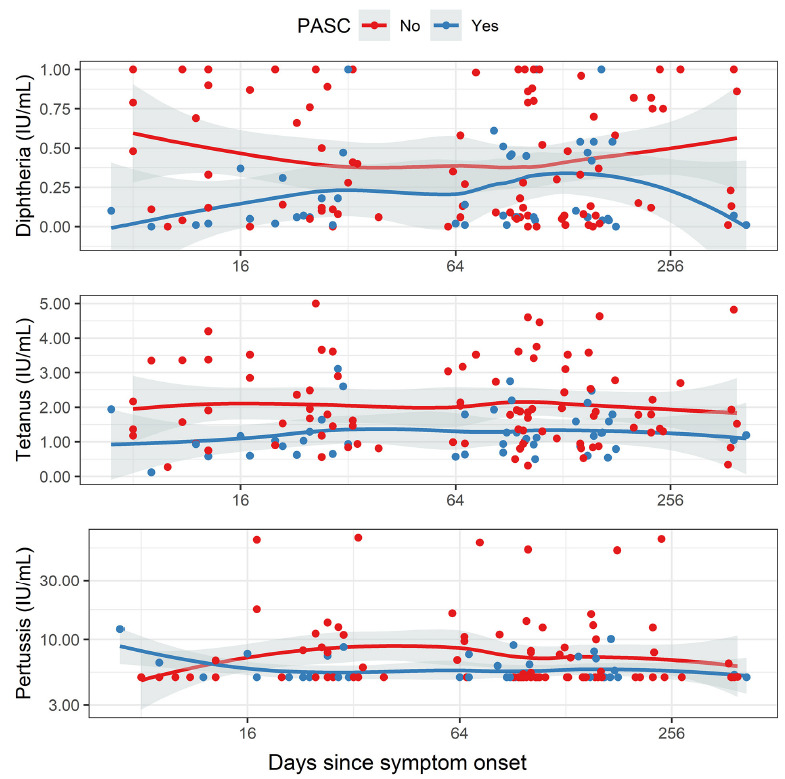
**Antibody levels plotted with time since symptom onset (days).** Red dots denote patients with no PASC (no impaired olfaction), and blue dots denote patients with PASC (impaired olfaction). Accordingly, the red line represents the local regression (as calculated with LOESS) in patients without PASC, and the blue line represents the moving average in patients with PASC.

## DISCUSSION

Our findings show no effect on unrelated vaccine antigen-specific antibody levels during or after SARS-CoV-2 infection, suggesting no decline in overall humoral immunity. This is also consistent with the lack of changes in the total IgG levels during infection or recovery. Anti-SARS-CoV-2 S protein IgG increased with time in the inpatients, but no increase was seen in the outpatients. Neutralizing antibodies against SARS-CoV-2 did not show differences between the disease phases; however, we did not have enough late samples to conclude this for inpatients, only for outpatients. The apparently stable COVID-19 antibody levels in mild COVID-19 cases can likely be attributed to the relatively late primary sampling of outpatients (Table 1). Primary sampling had to occur after the initial quarantine, and patients presumably had already developed a robust seroresponse at that time. In our cohort, the IgG levels against SARS-Cov-2 S protein increased for inpatients from the convalescent phase to the recovered phase, while in outpatients, the levels remained stable. A possible explanation for this could be that the inpatients, being older and presumably having more comorbidities, had already received the first doses of SARS-CoV-2 vaccines before the last sampling. Many of the last samples were taken in the spring of 2021, when the mass-vaccination campaign was initiated in Finland, first for the elderly and high-risk groups. To our knowledge, at least 6 patients had received one SARS-CoV-2 vaccine dose before their last sampling, but unfortunately, we were not able to obtain this data on all the study patients.

Our anti-vaccine antibody findings contradict some previous studies. It has previously been reported that there is an inverse correlation between anti-mumps IgG titers and COVID-19 severity [6], and anti-*Pneumococcus* IgG titers and COVID-19 severity [7]. Additionally, a positive correlation has been suggested between *Bordetella pertussis* antibody titers and COVID-19 disease severity [7]. A possible explanation for these findings is that certain risk factors of severe COVID-19 (such as obesity and advanced age) may also be risk factors for less effective immune responses to vaccinations [19]. When comparing COVID-19 patients with healthy controls, antibody titers against Rubella, pneumococcus, and *Bordetella pertussis* were found to be significantly lower in the patients [7]. Additionally, in patients who have recovered from COVID-19 versus healthy controls, measles-specific IgG was found to be significantly higher and tetanus antibody titers lower in patients [8]. As can be seen, these results also contradict each other.

Comparisons between the subgroups of inpatients and outpatients showed lower total IgG levels for inpatients in the acute disease phase. Furthermore, the results of SARS-CoV-2 S protein IgG and neutralizing antibody analyses suggested that more severe disease induces a more robust antibody response. There were no differences between the inpatient and outpatient groups in the diphtheria, tetanus, or pertussis toxoid IgG levels during the follow-up period. These results concur with previous data showing that low total IgG levels are associated with severe infections [20]. The kinetics of SARS-CoV-2 seroresponses for the most part matched earlier research [21].

A subanalysis of those patients who self-reported post-acute sequelae of COVID-19 (PASC+) and those who did not (PASC-) showed significantly lower anti-tetanus IgG in the acute and convalescent phases, and lower anti-diphtheria IgG in the acute phase. The PASC+ group was, on average, younger than the PASC− group, and it is possible that the timing of the latest vaccinations could explain the differences observed. According to the national vaccination program, individuals under the age of 65 years are vaccinated every 20 years with the diphtheria-tetanus booster (dT), while older individuals are boosted every 10 years. However, no differences in anti-diphtheria or anti-tetanus IgG were observed in the recovered phase, which would be expected if the vacci-nation schedule was the cause. Moreover, the anti-vaccine IgG levels did not correlate with age. The total IgG, anti-Bordetella pertussis, and anti-SARS-CoV-2 antibody levels were comparable between the individuals who reported prolonged symptoms and those who did not, which indicates that the PASC symptoms were not associated with overall weaker immune responses in our cohort.

We selected impaired olfaction as a representative of PASC symptoms because, together with impaired sense of taste, it was the only symptom that was more common among the SARS-CoV-2-positive than -negative individuals in our master study [16]. Moreover, unlike more generic symptoms, such as fatigue, it is uncommon among SARS-CoV-2-negative individuals [17, 18]. Our groups (PASC+/-) were not significantly different in sex distribution, disease severity, or length of hospitalization. In our study PASC+ patients were also younger than PASC− patients, contrary to previous studies [22, 23]. This could be due to our conservative definition of PASC or due to selection bias due to the small sample size.

Compared to previous studies, the present one had the benefit of sampling both in the acute phase and during a follow-up period (up to a year after infection). The effects of COVID-19 on routine vaccine antibody levels have not been previously investigated on such a long timescale. The 3-sample study design would have been able to show not only linear but also V- or inverted V-shaped trends. Another unique feature in the study design was the inclusion of mainly COVID-19 outpatients. This allows for the results to be generalized to mild COVID-19 cases, which form the majority of all cases, instead of the more severe hospital-treated population. To our knowledge, this is the first study of its kind to focus mainly on outpatients. Conversely, there were fairly few inpatients, and thus because of low statistical power, we cannot exclude that there may be a small effect in hospitalized patients that is not detected.

Another limitation of this study is the unequal sampling times. Late primary sampling of outpatients was necessary due to quarantine requirements (at the time 14 days from symptom onset). However, we believe that this does not affect the results since IgG-class antibodies have a half-life of about 25 to 30 days [24], which matches approximately with the boundary between the acute and convalescent disease phases (35 days). For the purposes of this study, we have considered the recovered phase (>120 days from symptom onset) to represent the baseline serology. However, we acknowledge the possibility of very long-lasting effects of COVID-19 on the antibody levels, in which case the recovered phase would not be equivalent to baseline. Accordingly, while none of the patients tested positive for COVID-19 reinfection during the follow-up period, there is a small possibility of symptom-free reinfection or of the patients not seeking an RT-PCR test for their symptoms. Another potential caveat is the unknown amount of time since the last booster vaccination of each patient. In the Finnish system, data on vaccinations are not stored centrally; thus, we were unable to obtain this data.

In conclusion, this study's main finding of no humoral immunity decline during or after COVID-19 infection is an encouraging one. Fortunately, our initial hypothesis of measles-like immune amnesia was proven incorrect. These findings, therefore, do not support any new recommendations in the treatment or follow up of COVID-19 patients. While it cannot be concluded that COVID-19 causes no changes to immunity, the present study adds to the current knowledge of the immune effects of COVID-19. The significance of the findings of lower anti-tetanus and anti-diphtheria IgG levels in patients that have impaired olfaction after COVID-19 remains unclear, yet there was no indication of overall weaker immune responses. Further research with larger sample sizes and a focus on severe COVID-19 is recommended.
